# An Aberrant Case of Large Submandibular Gland Sialolith With Obstructive Sialadenitis in a Young Female

**DOI:** 10.7759/cureus.44082

**Published:** 2023-08-25

**Authors:** Wajid Attar, Suresh Phatak, Avinash Dhok, Sandip Dhote, Kajal Mitra

**Affiliations:** 1 Radiodiagnosis, N. K. P. Salve Institute of Medical Sciences and Research Center and Lata Mangeshkar Hospital, Nagpur, IND

**Keywords:** submandibular gland abscess, submandibular swelling, submandibular gland calculus, sialadenitis, sialolithiasis

## Abstract

Sialolithiasis is a major cause of salivary gland dysfunction. Submandibular sialolithiasis with obstructive sialadenitis is a relatively uncommon condition. Submandibular gland sialolithiasis with obstructive sialadenitis accounts for about 2% of all cases of sialadenitis. We are reporting a case of a 20-year-old female who presented with acute-onset swelling in the right submandibular region. The submandibular gland had a large calculus with pericalcular abscess development, which was clearly visible on pre- and post-contrast computed tomography. Subsequently, surgical excision of the lesion was performed, and calculus was removed.

## Introduction

The formation of calculus in the salivary gland is known as sialolithiasis. Calculus is present in the parenchyma or duct leading to obstructed drainage of the salivary gland. This leads to stagnation and the formation of an abscess as a secondary infection supervenes [1]. The usual age group involved is 30-60 years; this condition is more commonly seen in males than females. The clinical presentation is usually seen as severe pain and enlargement of the gland. The degree of duct blockage is inversely correlated with pain intensity [2].

The etiopathogenesis of this condition is not clear. Organic and inorganic substances are seen within calculi including, phosphates, carbonate, glycoproteins, mucopolysaccharides, and cellular debris [2]. Other factors involved are less fluid intake and less saliva production as a result of pharmacological side effects. Tobacco chewing is also a potential risk factor described recently. It causes inflammation of salivary ducts and reduces levels of amylase causing sialolithiasis [3]. Different treatment options may be selected according to the size and location of the sialolith [4].

## Case presentation

**Figure 1:**
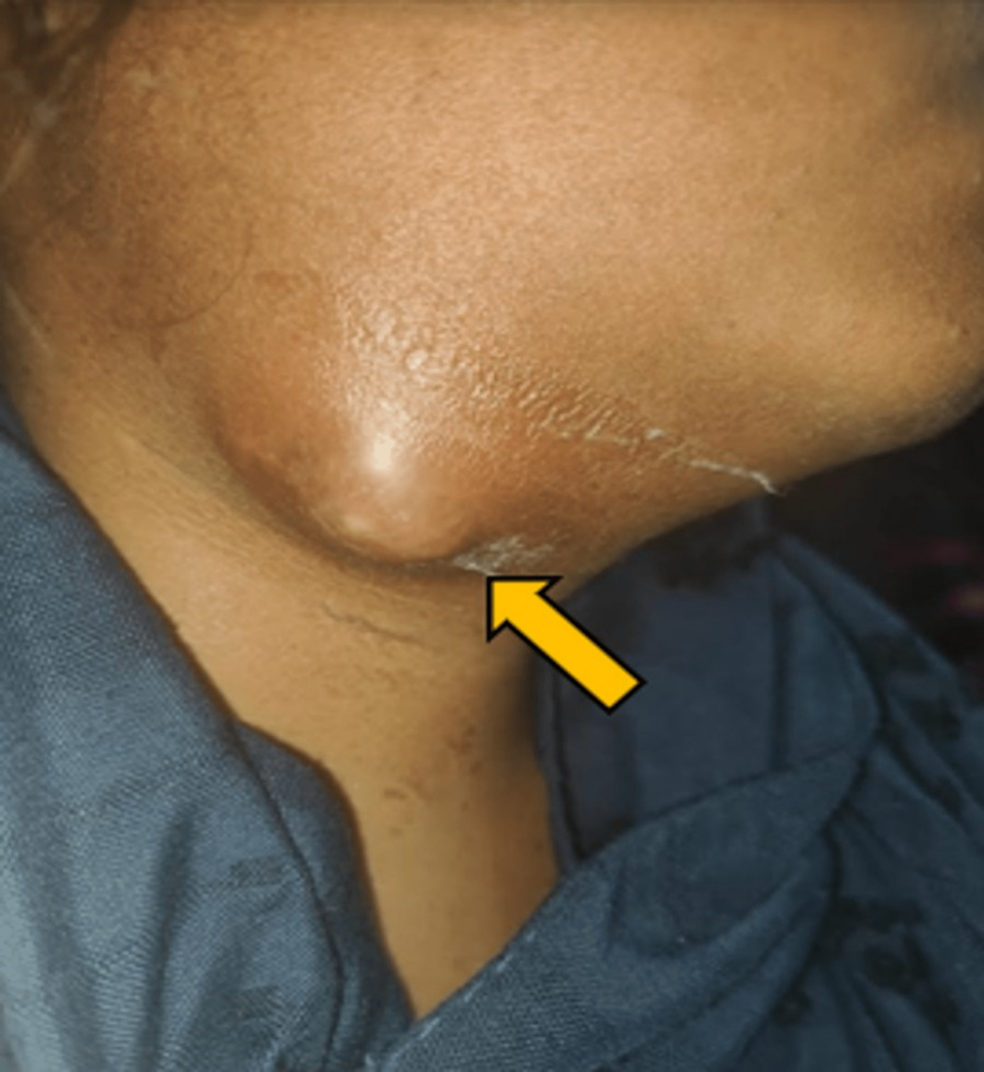
Swelling in the right submandibular region with erythema with raised local temperature and discharge (yellow arrow).

A 20-year-old female presented with swelling and tenderness in the submandibular region on the right side for 15 days (Figure 1).

**Figure 2:**
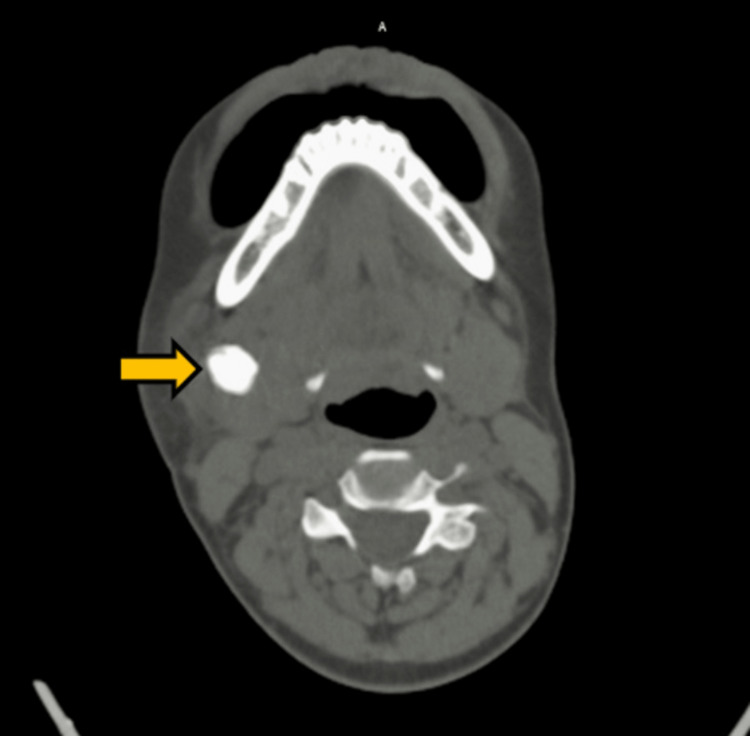
Axial pre-contrast CT of the neck at the level of the floor of the mouth demonstrating an ovoid calculus (yellow arrow) on the right of the midline. CT: computed tomography.

She reported intermittent swelling and pain during meals and an unpleasant taste while chewing food, which used to resolve after meals. On clinical examination, there was swelling in the submandibular region on the right side, visible extraorally. The overlying skin shows erythema and a raised local temperature. The patient had pus discharge from the submandibular region, where sialolith was found on imaging. Later blood investigations of the patient showed a raised total leukocyte count, which indicated infection surrounding the sialolith in the submandibular gland, which helped in the diagnosis. Axial pre-contrast computed tomography (CT) of the neck at the level of the floor of the mouth demonstrated an ovoid calculus of size 21 × 16 × 15 mm on the right side (Figure 2).

**Figure 3:**
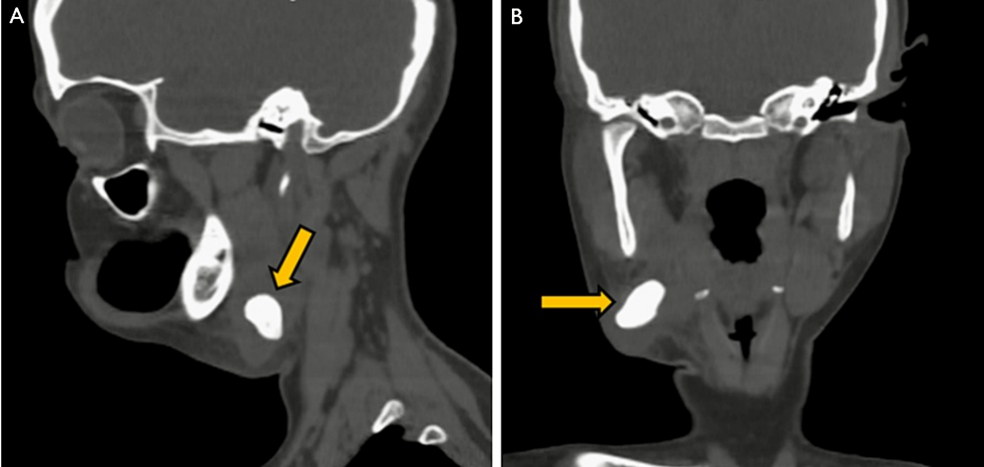
Sagittal pre-contrast CT image (A) at the level of the submandibular gland demonstrating calculus (yellow arrow). Coronal pre-contrast CT image (B) at the level of the submandibular gland demonstrating calculus (yellow arrow) on the right side. CT: computed tomography.

Sagittal and coronal pre-contrast CT sections at the right submandibular gland level demonstrated calculus (Figures 3A, 3B).

**Figure 4:**
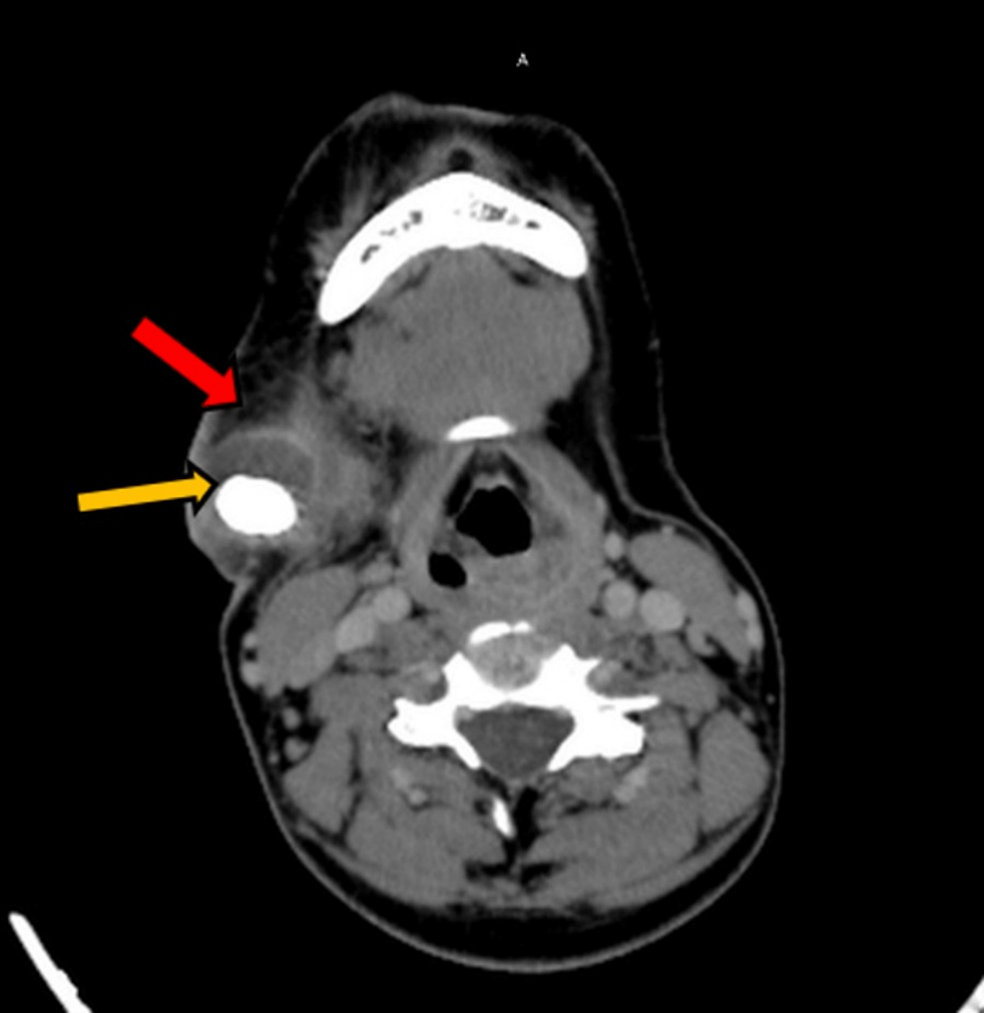
Axial CECT (post-contrast) image of the neck at the level of the floor of the mouth demonstrates calculus (yellow arrow) on the right side with a hypodense area around it (red arrow) representing abscess formation and inflammation in the submandibular gland. CECT: contrast enhanced computed tomography.

An axial contrast enhanced computed tomography (CECT) (post-contrast) image of the neck at the level of the floor of the mouth demonstrated calculus with a surrounding hypodense area of size 23 × 12 × 24 mm, representing abscess formation and inflammation in the submandibular gland (Figure 4).

**Figure 5:**
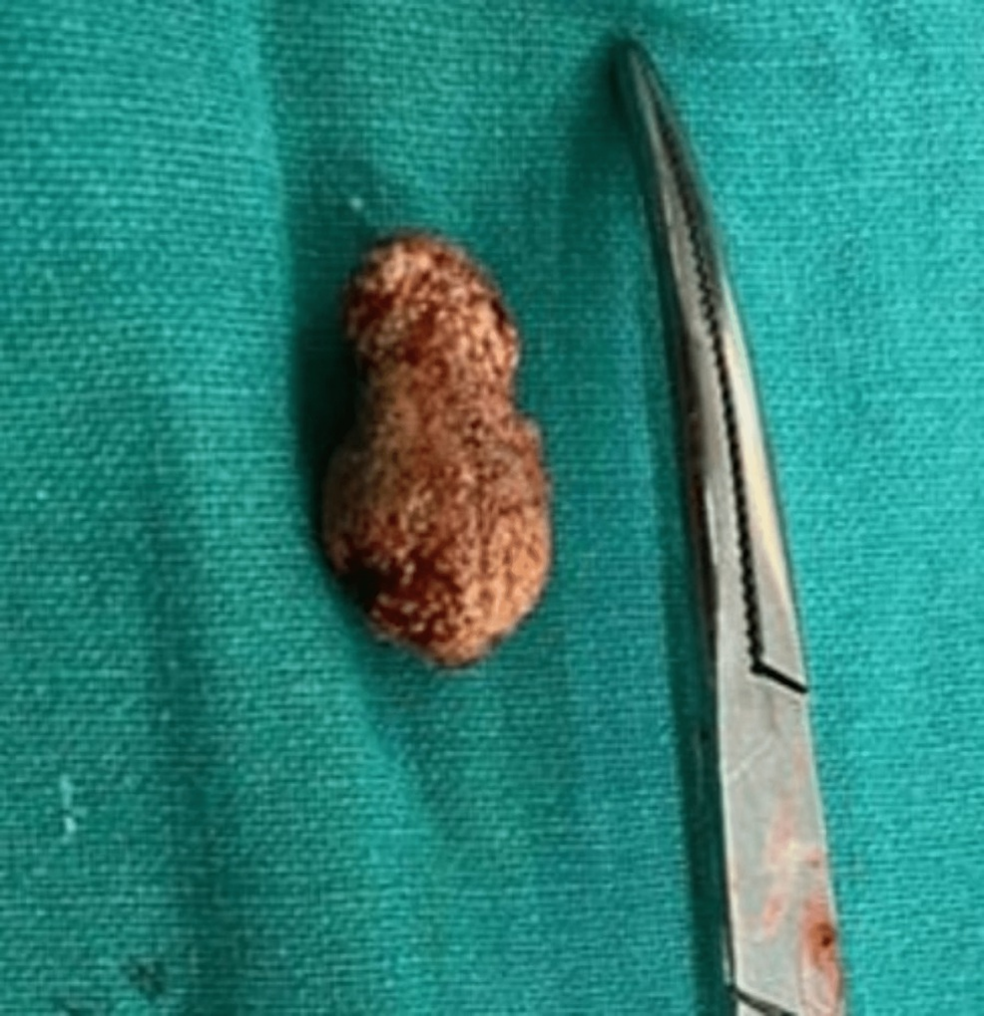
Post-operative submandibular gland excised sialolith.

The diagnosis was confirmed on CECT. The patient was well managed with surgical excision, and the stone was removed (Figure 5).

The patient did not develop postoperative complications, as seen on regular follow-up.

## Discussion

The anatomical location of the submandibular gland is within the submandibular triangle formed in between the digastric muscle and the mandible inferiorly. It consists of two lobes: the superficial lobe, which is the larger one, and the deep lobe. The incidence of sialolithiasis is highest in the submandibular gland, followed by the parotid and sublingual glands. The cause of maximum involvement of the submandibular gland is due to a long and irregular duct with a small diameter. Imaging studies are mandatory for the diagnosis of calculus disease [1]. Most of the calculi can be visualized on conventional radiography, but a few that are not heavily mineralized cannot be visualized on conventional radiography. So computed tomography (CT) and ultrasound are preferred for diagnosis. Radiographs taken in an oblique view are needed [5]. The role of ultrasound is established in calculus disease. Stones are seen as hyperechoic foci with or without acoustic shadowing posteriorly. Tiny calculi (less than 2 mm) may not demonstrate shadowing posteriorly. USG can catch radiolucent calculi as well. In the acute obstructive phase, an enlarged gland is seen along with a dilated duct proximal to the calculus [6,7]. A CT scan is the modality of choice to see calculi within the duct as well as the gland. In acute obstruction, an MRI reveals an enlarged gland associated with fat stranding and enhancement after gadolinium. MRI delineates ductal anatomy very well. All sequences demonstrate stones as low signal intensity outlined by high signal intensity on T2 weighted image (WI). In the case of nonobstructive calculus, the gland appears completely normal on MRI [8,9].

After treatment of calculus disease, the patient is advised to take a diet that is rich in protein, liquid, and acidic foods to avoid calculi formation again. Various differential diagnoses of calculus disease include salivary gland tumors, mucoceles, and mucus cysts [10].

## Conclusions

Submandibular sialolithiasis in a young female is an uncommon condition, and usually, the patient presents with severe pain and swelling. Preoperative clinical and imaging examinations are important for accurate diagnosis and treatment. CT is a well-established modality for the early and accurate diagnosis of sialolithiasis, helping in patient management and preventing further complications.
